# Innovative biomarkers to predict unfavourable outcomes after initiating multiple sclerosis treatment

**DOI:** 10.1007/s00415-022-11300-x

**Published:** 2022-08-01

**Authors:** Karim L. Kreft, Neil P. Robertson

**Affiliations:** 1 Department of Neurology, University Hospital Wales, Cardiff, CF14 4XN UK; 2 Institute of Psychological Medicine and Clinical Neuroscience, Cardiff University, University Hospital of Wales, Heath Park, Cardiff, CF14 4XN UK

Over the last decade, more than ten new disease modifying treatments (DMTs) for multiple sclerosis (MS) have been approved and introduced into routine clinical care. All have demonstrated improved efficacy in limiting ongoing inflammation and tissue damage in MS patients, but many have potentially serious side effect profiles that require more careful monitoring than previous baseline therapies. Together with the tendency for earlier introduction of high efficacy DMTs, the risk that patients will encounter adverse events will increase over time. Identifying biomarkers which enable accurate prediction of prognosis and treatment response has become a major challenge to those working in the field. In this journal club, we describe three novel studies aiming to predict and detect serious side effects associated with MS treatments.

## Deep immunophenotyping using mass cytometry and supervised machine learning to predict dimethyl fumarate-associated lymphopenia

Dimethyl fumarate (DMF) acts on both T- and B-lymphocytes and myeloid cells via the Nrf2 pathway to inhibit inflammation. Lymphopenia is an established adverse event, associated with an increased risk of progressive multifocal leukoencephalopathy (PML); a potentially life-threatening opportunistic infection caused by the JC polyomavirus. Strategies identifying patients at risk of developing lymphopenia are therefore essential in guiding clinical management. This study by Diebold et al. developed a deep phenotyping strategy using high-dimensional mass cytometry and supervised machine learning to establish suitable biomarkers for the development of DMF-associated lymphopenia. Thirty-one MS patients were followed prospectively from baseline with total lymphocyte counts and serial flow cytometry using 36 immune-related markers. Ten patients developed lymphopenia. Using the CellCNN supervised machine learning algorithm, rare cell populations associated with lymphopenia were identified. In all MS patients, DMF most profoundly reduced T-cells; in individuals developing lymphopenia this was more pronounced. In lymphopenic patients, a relative increase in myeloid and B-cells was observed, whereas this phenomenon was absent in non-lymphopenic patients. The authors also assessed which cell type at baseline could predict lymphopenia during follow-up. Effector memory T-helper cells were decreased at all timepoints in lymphopenic patients and had a predictive value of approximately 74%.

Comment: This study identified a subpopulation of T-helper effector memory cells expressing CCR4, CD25, CD103 and IL10 as a marker for DMF-induced lymphopenia. This sub-population mimicks a regulatory T-cell phenotype with low expression of the Th17 cells considered pathogenic in MS. Interestingly, low levels of effector memory T-helper cells are also associated with fatal DMF-associated PML cases and therefore may serve as a relevant biomarker to guide clinical decisions. However, the study has limited participants and, as acknowledged by the authors, the findings need validation in an independent cohort.

Diebold M, Galli E (2022) Immunological predictors of dimethyl fumarate-induced lymphopenia. Ann Neurol. 

## Use of translocator protein PET imaging in natalizumab-associated progressive multifocal leukoencephalopathy

MS patients receiving natalizumab (an anti- α4-integrin monoclonal antibody inhibiting leukocytes migrating across the blood brain barrier) have an increased risk of developing PML. Early identification of PML is problematic as reliable imaging biomarkers are currently lacking and can have a similar appearance to MS lesions. In addition, interpretation of sequential MRI in natalizumab-associated PML can be challenging, especially in the context of rebound MS disease activity after natalizumab withdrawal.

The mitochondrial translocator protein (TSPO) is a non-specific marker of inflammation. Histopathological studies have demonstrated that TSPO is increased in both PML-associated brain lesions as well as in MS lesions. Mahler et al. investigated whether positron emission tomography (PET) could be advantageous compared with MRI in diagnosing and following the natural course of natalizumab-induced PML in MS patients.

The ^18^F-GE-180 PET tracer was used to bind to TSPO. Eight MS patients with PML underwent PET in parallel with 3 T MRI scans at several timepoints. They found that PET imaging was able to detect PML lesions at all time points, even before gadolinium enhancement was apparent on MRI. Interestingly, the tracer remained detectable some years after PML, indicating that it may be a suitable long-term follow-up marker. By applying several PET imaging criteria, including the appearance of the lesion and the tracer uptake pattern, the authors could correctly assign lesions as MS or PML with an accuracy of 96%.

Comment: This single-centre study has identified a potentially important new biomarker for natalizumab-induced PML. Further validation of their findings is important, especially to determine if their PET technique is also able to differentiate between similarly appearing small PML and MS lesions and also tumefactive MS lesions from large PML areas with inflammation.

Mahler C, Schumacher A-M (2021) TSPO PET imaging of natalizumab-associated progressive multifocal leukoencephalopathy. Brain. https://doi.org/10.1093/brain/awab127

## Ratio of B-lymphocytes/plasma cells predicts secondary auto-immunity after alemtuzumab treatment

Approximately one-third of the MS patients treated with alemtuzumab (anti-CD52 monoclonal antibody) will develop secondary autoimmunity, mainly thyroid disease. Alemtuzumab depletes both T- and B-lymphocytes with the latter repopulating earlier. When initiating treatment, patient-specific risk of secondary autoimmunity is unclear. Walo-Delgado et al. assessed whether baseline immunophenotyping could be employed to predict this risk in MS patients. In a multicentre prospective cohort study which included 59 MS patients, peripheral blood mononuclear cells were collected at baseline and in 39 at one year of treatment. All patients underwent routine screening for side-effects. Twenty-two patients developed a secondary auto-immune disease, of which twenty-one had thyroid autoimmunity. Baseline immunophenotyping differed between patients with and without secondary auto-immune diseases; those without secondary autoimmunity had lower T-helper cells (mainly effector memory and terminally differentiated T-cells) and TNF-α producing cytotoxic T-cells. In contrast, patients with a secondary autoimmunity had more B-cells at baseline, mainly immunoglobulin producing plasmablast/plasma cells and to a lesser extent naïve B-cells. These findings were validated following assessment of absolute cell counts. One year following first dose of alemtuzumab, the altered composition of lymphocytes remained present. An area under the curve (AUC) analysis of percentage of plasma cells/plasmablast at baseline had a predictive value of 0.75 for secondary autoimmunity.

Comment: This study highlights the importance of B-lymphocytes in development of auto-immunity. However, it should be noted that the plasma cell/plasmablast percentage cannot be used if patients received natalizumab prior to alemtuzumab, since natalizumab lowers the number of plasmablasts.

Walo-Delgado PE, Monreal E (2021) Role of B cell profile for predicting secondary autoimmunity in patients treated with alemtuzumab. Front Immunol. https://doi.org/10.3389/fimmu.2021.760546

